# Primary Pigmented Nodular Adrenocortical Disease Presenting as Osteonecrosis of the Femoral Head: A Case Report

**DOI:** 10.7759/cureus.108027

**Published:** 2026-04-30

**Authors:** Yunfeng Zhen, Hanlu Xiang, Cuijuan Qi, Guangyao Song

**Affiliations:** 1 Department of Endocrinology, Diabetes and Metabolism, Hebei General Hospital, Shijiazhuang, CHN; 2 Department of Neurology, Hebei Medical University, Shijiazhuang, CHN

**Keywords:** carney complex, cushing disease, femoral head necrosis, pde11a, primary pigmentary nodular adrenocorticopathy, unilateral adrenalectomy

## Abstract

Primary pigmented nodular adrenocortical disease (PPNAD) represents a rare etiology of adrenal-derived, adrenocorticotropic hormone (ACTH)-independent hypercortisolism. Its pathogenesis is primarily attributed to inactivating mutations in the *PRKAR1A*, *PDE11A*, or *PD88* genes, as well as amplifications of *PRKACA* and triploid mutations of *PRKACB*. This report presents the case of a patient with PPNAD, who exhibited symptoms including hypertension, femoral head necrosis, anxiety and depression, nephrolithiasis, and short stature. Genetic analysis revealed a heterozygous mutation in the *PDE11A* gene (NM_016953.4: c.2032G>A; Ala678Thr). Additionally, a review of recent literature was conducted to enhance clinicians' understanding of this condition.

## Introduction

The clinical presentation of Cushing's syndrome (CS) arises from long-term exposure to excessive cortisol, with key symptoms including a moon-shaped face, central obesity, purple streaks on the skin, facial redness, thin skin, acne, and increased body hair. Frequent causes of CS include pituitary adenomas, tumors producing ectopic adrenocorticotropic hormone (ACTH), adrenocortical carcinoma, or hyperplasia [1].

Primary pigmented nodular adrenocortical disease (PPNAD) is an uncommon cause of adrenal-origin, ACTH-independent hypercortisolism. This condition is more common in children and young adults and is marked by several functional pigmented nodules in the adrenal cortex, along with atrophy of the cortical areas between the nodules. Due to the small nodular formations (under 1 cm), radiology can underestimate the involvement of both adrenal glands in patients with PPNAD. These micronodules frequently produce cortisol and consist of cortical cells with low lipid content, leading to hypercortisolism in PPNAD that can be overt, subclinical, cyclic, or atypical. All these make the diagnosis of PPNAD difficult. Most patients with PPNAD or Carney complex have a familial history involving mutations in *PRKAR1A*, *PDE11A*, *PDE8B*, *PRKACA*, *ARMC5*, or β-catenin (*CTNNB1*) genes [1-3].

This report discusses and analyses the clinical presentation and genetic characteristics of a patient with PPNAD treated at our institution.

## Case presentation

A 25-year-old male patient was transferred to our hospital from an outside hospital on September 20 for further treatment. 

Seven months prior to this, the patient had developed left thigh soreness, leading to a diagnosis of avascular necrosis of the left femoral head four months later. For further diagnostic evaluation and management, he was admitted to a local hospital. The patient had a history of hypertension for more than five years and the development of purple striae over the past two years. He first noted elevated self-monitored blood pressure (BP) more than five years prior, with systolic levels reaching up to 140 mmHg; he had not been using antihypertensive medications or conducting regular BP monitoring. Two years before admission, the patient observed the appearance of purple striae on the abdomen, increased vellus hair, and an increase in abdominal circumference. Approximately one year before admission, his BP reached levels up to 180/120 mmHg, and treatment was initiated with oral reserpine and sustained-release nifedipine tablets, which maintained blood pressure at approximately 150/100 mmHg. Over the preceding three years, the patient experienced recurrent urinary calculi and underwent three lithotripsy procedures. He reported no history of glucocorticoid use since disease onset, and he has also had a two-year history of anxiety and depression. Notably, his height has been stable since the age of 15 years. The family history was non-contributory. Comprehensive laboratory tests were conducted at the outside hospital (Tables 1, 2).

**Table 1:** Screening for the cause of hypertension in the outside hospital RAAS: renin–angiotensin–aldosterone system; PRA: plasma renin activity; AT-II: angiotensin II; ALD: aldosterone; FSH: follicle-stimulating hormone; LH: luteinizing hormone; T: testosterone; PRL: prolactin; TSH: thyroid-stimulating hormone; TT4: total thyroxine; FT4: free thyroxine; FT3: free triiodothyronine; TT3: total triiodothyronine; DHEA-S: dehydroepiandrosterone sulfate; 17α-OHP: 17-hydroxyprogesterone

Exam Items	Patient Values	Reference Ranges
RAAS (Random upright）		
PRA	3.83 ng/ml/h	0.1–6.56
AT-Ⅱ	84.50 pg/ml	50–120
ALD	4.62 ng/dl	7–30
Sex Hormones		
FSH	5.03 mIU/ml	1.5–12.4
LH	1.56 mIU/ml	1.7–8.6
T	3.85 ng/ml	2.49–8.36
PRL	502.00 uIU/ml	86–324
Thyroid Function		
TSH	1.84 uIU/ml	0.35–0.45
TT4	121.00 nmol/L	64.35–167.31
FT4	18.50 pmol/L	12–24
FT3	5.68 pmol/L	3.5–7
TT3	1.92 nmol/L	1.23–2.92
DHEA-S	223.00 μg/dl	85–690
17α-OHP	2.432 ng/ml	0.312.01

**Table 2:** Qualitative positioning examination of Cushing syndrome in the outside hospital UFC: urinary free cortisol; LDDST: low-dose dexamethasone suppression test; HDDST: high-dose dexamethasone suppression test

Test/Sample Collection	Plasma Cortisol（8:00 am, reference range: 6.02–18.4)	Plasma Cortisol（0:00 am)	Serum ACTH （8:00 am, reference range: 7.2–63.3)	24-hour UFC (reference range: 30–350)
September 8	20.80 μg/dl	18.60 μg/dl	6.92 pg/mL	960 (1.6 L) μg/24h
September 10				600 (1 L) μg/24h
LDDST	21 μg/dl		2.08 pg/mL	840 (1.4 L) μg/24h
HDDST	15.01 μg/dl		3.78 pg/mL	1500 (2.5 L) μg/24h

Physical examination

On admission, the physical examination showed height 157 cm, weight 58 kg, body mass index (BMI) 23.53 kg/m², and waist circumference 88.5 cm. Observations include scattered ecchymosis across the body, multiple acne lesions on the trunk, prominent vellus hair, wide purple abdominal striae, supraclavicular fat pads, buffalo hump, abdominal obesity, thin limbs, patchy lip pigmentation, and blue nevi on the first toes of both feet.

Laboratory tests

**Figure 1:**
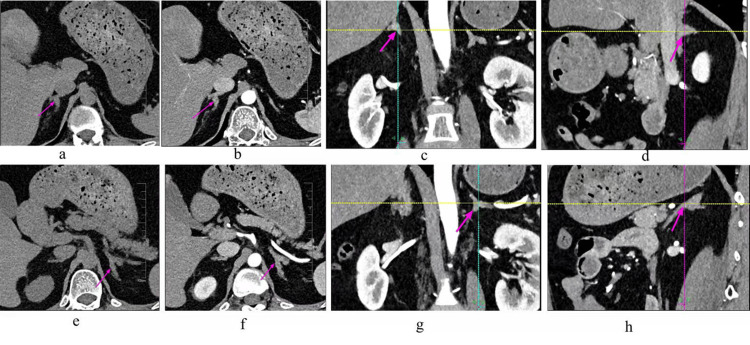
Bilateral adrenal CT scan with contrast enhancement (a) CT scan of the left adrenal gland.(b) Contrast-enhanced CT of the left adrenal gland. (c) Contrast-enhanced CT of the left adrenal gland in the coronal plane. (d) Sagittal CT with contrast of the left adrenal gland. (e) CT scan of the right adrenal gland. (f) Contrast-enhanced CT of the right adrenal gland. (g) Contrast-enhanced CT of the right adrenal gland in the coronal plane. (h) Sagittal CT with contrast of the right adrenal gland.

Routine biochemistry, assessment of complications associated with Cushing syndrome, and Carney complex screening are shown in Table 3. Cushing’s syndrome evaluation showed autonomous cortisol secretion (Table 4). Bone mineral density is shown in Table 5. Bilateral adrenal CT scans with contrast enhancement demonstrated bilateral adrenal nodular hyperplasia (Figure 1). A non-contrast MRI of the pituitary gland showed no abnormalities. Urinary ultrasound identified bilateral kidney stones, calcified plaques in the prostate, and no significant dilation of the ureters. Cervical vascular ultrasound showed no significant abnormalities. Scrotal, testicular, and epididymal color ultrasound examinations were normal. Thyroid ultrasound was unremarkable.

**Table 3:** Routine biochemistry in our hospital HDL-C: high-density lipoprotein cholesterol; LDL-C: low-density lipoprotein cholesterol; FBG: fasting blood glucose; HbA1c: glycated hemoglobin, SaO2: oxygen saturation of arterial blood

Exam Items	Results	Reference Range
Serum potassium	3.3 mmol/L	3.5-5.3
Serum sodium	144 mmol/L	137-147
Serum creatinine	61 umol/L	50–120
24-hour urine potassium	26.18 mmol	
Serum total cholesterol	5.89 mmol/L	3.1-5.2
Serum triglycerides	2.24 mmol/L	0.4-1.82
HDL-C	1.23 mmol/L	
LDL-C	3.85 mmol/L	
FBG	3.68 mmol/L	3.9-6.1
HbA1c	5.9%	4.0%-6.0%
D-dimer	0.42 mg/L FEU	0-0.55
Blood gas analysis		
PH	7.43	12–24
SaO2 (routine air)	97.67mmHg	3.5–7
Actual bicarbonate	25.8 mmol/L	22-27
Base excess	1.56 mmol/L	
Bone metabolism		
Parathyroid hormone	26.00 pg/mL	15–57
25-hydroxyvitamin D	41.50 ng/mL	20–100
Total procollagen I N-terminal peptide	49.34 ng/mL	32–89.1
β-C-terminal telopeptide of type-Ⅰ collagen	0.95 ng/mL	0.029–0.585
24-hour urinary calcium	5.19 mmol	
24-hour urinary phosphorus	23.23 mmol	
Carney complex screening		
Growth hormone	0.501 ng/mL	0.06–5.0
Insulin-like growth factor 1	219.6 ng/mL	60–350
Prolactin	27.51 ng/mL	4.04–15.2
Calcitonin	2.09 pg/mL	1.01–1.2
Carcinoembryonic antigen	2.41 ng/mL	＜5

**Table 4:** Qualitative positioning examination of Cushing syndrome in the our hospital

Test/Sample Collection	Plasma Cortisol（8:00 am, 6.02–18.4μg/dl)	Plasma Cortisol（0：00, μg/dl)	Serum ACTH （8:00 am, pg/mL)	24-hour UFC (μg/24h, 30–350)
2023-9-8	17.96	15.03	1.42	840 (1.8 L)
2023-9-10				600 (1 L)
LDDST	21		2.08	840 (1.4 L)
HDDST	15.01		3.78	1500 (2.5 L)

**Table 5:** Bone mineral density (BMD) THTOAL: total bone density of the left hip joint

Location	BMD（g/cm^2^）	Z-value
L1	0.655	–3.2
L2	0.795	–2.7
L3	0.734	–3.4
L4	0.761	–3.5
L1-L4	0.738	–3.2
Left hip neck	0.693	–1.7
THTOAL	0.647	–2.6

Whole-exome sequencing (WES)

WES identified a point mutation in *PDE11A* (NM_016953.4: c.2032G>A; Ala678Thr) on chromosome 2. The genetic defect was associated with PPNAD type 2, a rare form of ACTH-independent Cushing syndrome. The clinical phenotype associated with the *PDE11A* mutation was investigated using the Online Mendelian Inheritance in Man (OMIM) database (https://www.omim.org/), confirming an autosomal dominant inheritance pattern. A search of the ClinVar database (https://www.ncbi.nlm.nih.gov/clinvar/) for mutations at this locus revealed no functional validation. The American College of Medical Genetics and Genomics (ACMG) variant classification [4] indicated a variant of uncertain significance. Predictive analyses yielded mixed results: (i) PolyPhen-2 predicted the mutation as probably damaging (score 0.958; sensitivity 0.78; specificity 0.95) [5], (ii) SIFT (Sorting Intolerant from Tolerant) predicted minimal impact on protein function (score 0.44) [6], and (iii) Rare Exome Variant Ensemble Learner (REVEL) suggested probable pathogenicity (score 0.592) [7]. Confirmation of PPNAD via adrenal biopsy and histopathological analysis was not performed, as subsequent endocrinological evaluations revealed normal values, and the patient's symptoms improved postoperatively. Further investigation is warranted to clarify the role of the *PDE11A* mutation in the pathogenesis of PPNAD in this patient.

Family history

**Figure 2:**
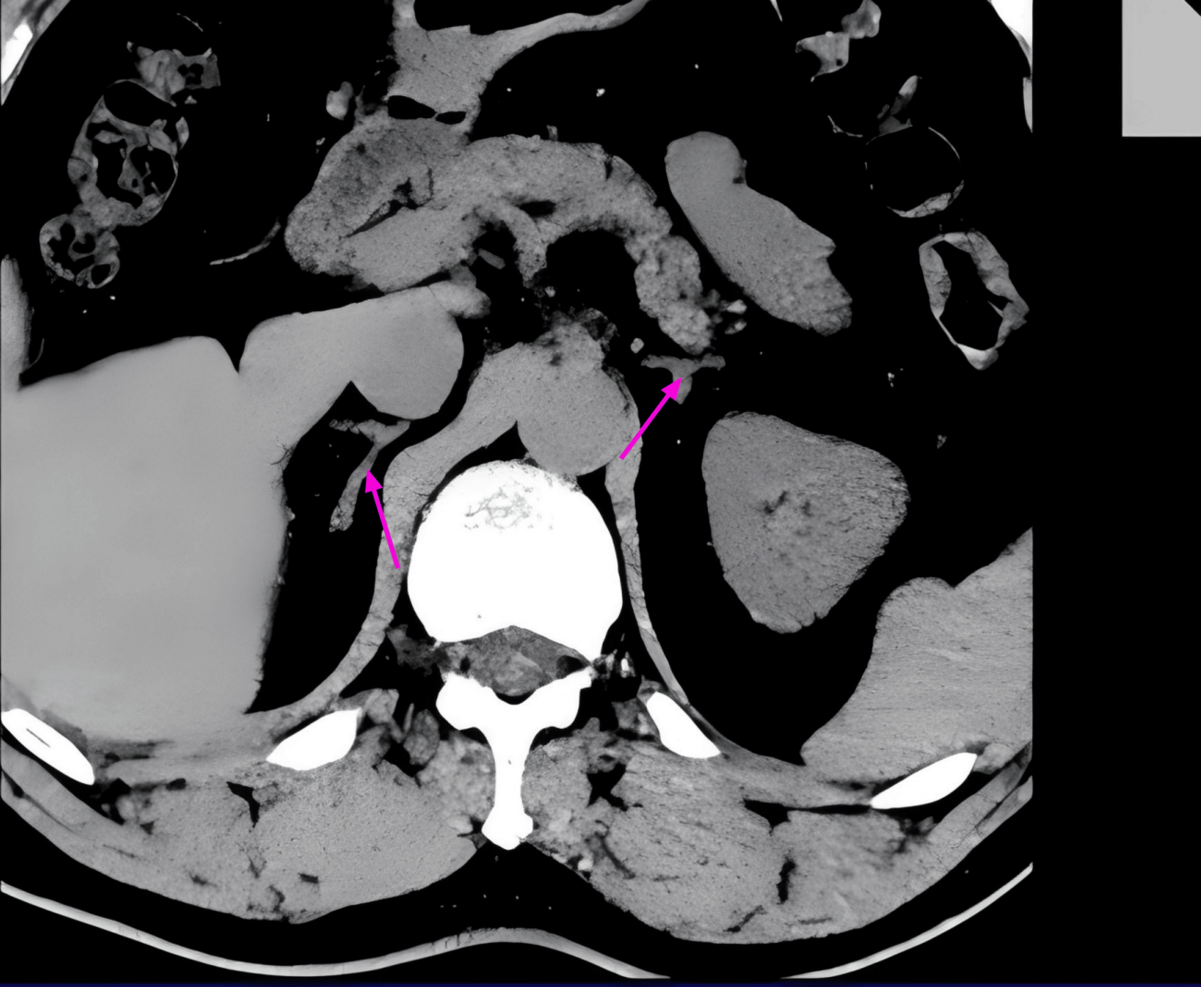
Non-contrast CT scan of the patient's father's adrenal glands

Analysis of the variant in the patient's parents revealed that the mutation was inherited from the father and was absent in the mother. The father occasionally experienced elevated BP during episodes of insomnia, with a maximum BP of 140/90 mmHg, without the use of antihypertensive medication; his usual BP ranged from 110-120/60-80 mmHg. Physical examination of the father revealed no signs of typical Cushing's facies, no blue nevi on the skin or mucous membranes, and pigmentation of the left auricle. Non-contrast CT scans of the father's adrenal glands showed both glands to be generally normal, with no nodular changes (Figure 2). The father's laboratory values included serum ACTH (8:00 am), 53.74 pg/mL, plasma cortisol (8:00 am), 6.98 µg/dL, and 24-hour urinary free cortisol (UFC), 214.08 µg (reference 30-350).

Diagnosis and treatment

**Figure 3:**
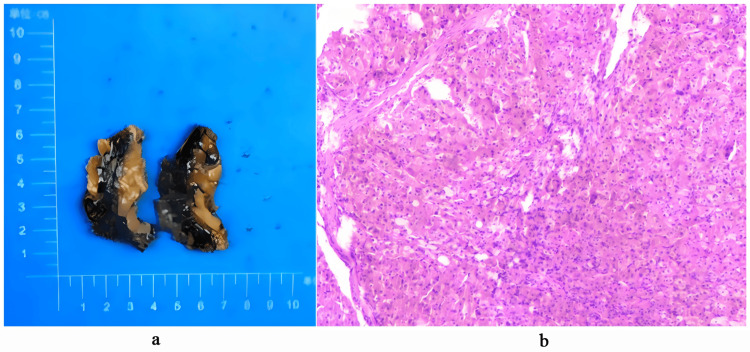
Adrenal gland pathology of the patient (a) Appearance of the adrenal gland. (b) Hematoxylin and eosin staining.

The diagnosis of PPNAD was confirmed based on the patient's characteristic Cushingoid appearance, supplementary examinations, and genetic testing. Carney complex could not be definitively excluded. Traditionally, bilateral adrenalectomy has been the primary treatment for overt Cushing syndrome and adrenal hyperplasia and is commonly chosen for adult patients with PPNAD. However, unilateral adrenalectomy may be a viable option for patients with PPNAD to reduce the risk of permanent adrenal insufficiency, particularly in younger individuals [8,9]. In this patient, the decision to perform unilateral surgery was based on the relative size of the adrenal glands and the surgeon's observations during the operation [10]. The left adrenal gland was notably larger than the right and contained several large nodules (Figure 3a); therefore, laparoscopic resection of the left adrenal mass was performed. Postoperative pathology confirmed adrenal cortical nodular hyperplasia, measuring 5 × 3 × 1 cm, with a gray-yellow appearance (Figure 3b).

Postoperatively, the patient received short-term hydrocortisone therapy. Follow-up plasma cortisol (8:00 am) was 12.44 μg/dL, indicating no significant adrenal insufficiency, and glucocorticoid replacement therapy was gradually discontinued. The patient maintained a good appetite and physical strength, lost 5.5 kg, and exhibited mild abdominal skin peeling. One year after surgery, laboratory values were: serum ACTH (8:00 am), 3.12 pg/mL, plasma cortisol (8:00 am), 8.37 µg/dL, 24-hour UFC, 198.29 µg. Classic low-dose dexamethasone suppression test (LDDST) results showed plasma cortisol (8:00 am) was 1.71 µg/dL and 24-hour UFC was 20.37 µg, suggesting substantial improvement of autonomous cortisol secretion after unilateral adrenalectomy.

## Discussion

This case study presents a patient diagnosed with ACTH-independent Cushing's syndrome, characterized by adrenal CT revealing nodular hyperplasia. WES identified a mutation in the *PDE11A* gene. Following unilateral adrenalectomy, the patient experienced remission of hypercortisolemia, culminating in a diagnosis of PPAND in correlation with the observed adrenal pathology.

To date, six distinct gene mutations have been associated with PPNAD: *PRKAR1A*, *PRKACA*, *PDE11A*, *PDE8B*, *ARMC5*, and *CTNNB1*. Certain cases of corticotropin-independent macronodular adrenal hyperplasia often exhibit inactivating *ARMC5* mutations. A significant correlation has been observed between *PRKAR1A *mutations and spotty skin pigmentation in classic PPNAD [11]. Multiple case reports have documented *PDE11A* mutations in isolated PPNAD or Carney complex, often linked to adrenal tumors. Functional analyses exist for some *PDE11A* variants but are not comprehensive [12,13]. Horvath et al. demonstrated elevated cAMP levels in human embryonic kidney 293 and mouse Leydig tumor cell line cells following the overexpression of *PDE11A* variants (p.Arg52Thr, p.Phe258Tyr, p.Gly291Arg, p.Ala349Thr, p.Asp609Asn, p.Tyr727Cys, p.Val820Met, p.Arg804His, p.Arg867Gly, and p.Met878Val) [14]. Studies indicate that males with *PDE11A* variants have a higher propensity than females to develop PPNAD [15-17].

Genetic testing in this patient revealed a heterozygous *PDE11A* mutation (NM_016953.4: c.2032G>A; Ala678Thr). While its pathogenic role in this patient is not confirmed, the mutation resides in the catalytic domain of the PDE11A protein and has the potential to alter its enzymatic activity or interactions with other proteins [18]. Wang et al. (2019) reported the only other case involving the same PDE11A mutation; however, their patient demonstrated paraneoplastic ACTH secretion due to a lung carcinoid, which was resolved following surgical intervention [19].

PolyPhen-2 and REVEL predict likely pathogenicity. Despite the lack of functional validation in the patient, the combination of typical clinical manifestations, adrenal CT imaging, postoperative appearance, and pathology of the adrenal glands, and WES findings supports a pathogenic role. Screening for the Carney complex revealed lip pigmentation, blue nevi on both first toes, normal growth hormone (GH) and insulin-like growth factor 1 (IGF-1) levels, and normal cardiac, thyroid, and testicular ultrasounds, indicating that Carney complex could not be definitively excluded.

*PDE11A* encodes a phosphodiesterase responsible for regulating cAMP signaling in adrenocortical cells, and loss-of-function variants in this gene result in overactivation of the cAMP-PKA pathway, leading to excessive cortisol production and adrenocortical hyperplasia associated with Cushing syndrome [20]. In the present patient, the proband harboring the *PDE11A* pathogenic variant presented with typical clinical features of Cushing syndrome, whereas his father, carrying the identical variant, was asymptomatic, demonstrating incomplete penetrance and remarkable phenotypic heterogeneity of *PDE11A*-related adrenal diseases. This phenotypic discordance can be attributed to compensatory effects of other phosphodiesterase isoforms, sex-specific hormonal environments, age-related epigenetic modifications, and individual genetic modifier backgrounds, indicating that the *PDE11A* variant alone is not sufficient for disease onset and that the clinical phenotype is determined by the interplay of genetic, hormonal, and environmental factors [21,22]. Vaduva et al. (2025) found that patients with damaging variants in both *PDE11A* and *ARMC5* exhibit a less severe phenotype, including lower UFC levels, reduced midnight plasma cortisol concentrations, and fewer adrenal nodules compared with those harboring only *ARMC5* variants [23]. This suggests that *PDE11A *may modulate the phenotype of PBMAH, potentially informing patient management strategies. The patient's father carries the *PDE11A* mutation but exhibited no hypercortisolism symptoms; blood pressure, plasma cortisol, and serum ACTH levels were within normal ranges. Careful monitoring is nevertheless recommended in such cases too.

## Conclusions

This case substantiates the *PDE11A* c.2032G>A mutation as the etiological factor for PPNAD, with WES facilitating precise diagnosis of this uncommon form of ACTH-independent hypercortisolism. The successful resolution of postoperative hypercortisolism following laparoscopic resection of the left adrenal mass underscores the effectiveness of surgical intervention in managing this rare condition.
